# Sub-Nanosecond Dynamics of Pathologically Relevant Bio-Macromolecules Observed by Incoherent Neutron Scattering

**DOI:** 10.3390/life12081259

**Published:** 2022-08-17

**Authors:** Tatsuhito Matsuo, Judith Peters

**Affiliations:** 1 Institute for Quantum Life Science, National Institutes for Quantum Science and Technology, 2-4 Shirakata, Tokai 319-1106, Ibaraki, Japan; 2 Dept. of Physics, Univ. Grenoble Alpes, CNRS, LiPhy, 38000 Grenoble, France; 3 Institut Laue-Langevin, 71 Avenue des Martyrs, CEDEX 9, 38042 Grenoble, France; 4 Institut Universitaire de France, 75231 Paris, France

**Keywords:** incoherent neutron scattering, molecular dynamics, protein, lipid, human disease

## Abstract

Incoherent neutron scattering (iNS) is one of the most powerful techniques to study the dynamical behavior of bio-macromolecules such as proteins and lipid molecules or whole cells. This technique has widely been used to elucidate the fundamental aspects of molecular motions that manifest in the bio-macromolecules in relation to their intrinsic molecular properties and biological functions. Furthermore, in the last decade, iNS studies focusing on a possible relationship between molecular dynamics and biological malfunctions, i.e., human diseases and disorders, have gained importance. In this review, we summarize recent iNS studies on pathologically relevant proteins and lipids and discuss how the findings are of importance to elucidate the molecular mechanisms of human diseases and disorders that each study targets. Since some diseases such as amyloidosis have become more relevant in the aging society, research in this field will continue to develop further and be more important in the current increasing trend for longevity worldwide.

## 1. Introduction

Proteins and lipids are bio-macromolecules essential for life, together with nucleic acids [1]. Proteins are abundant molecular species, which account for ~50% of the cell’s dry mass [2]. While some proteins serve as a structural component of the cell, other proteins execute almost all the cellular functions. Lipid molecules are the main components of bio-membranes, which define the boundaries between the interior and the exterior of the cell, or between the inside and the outside of organelles. In addition to this structural role, lipid membranes, into which membrane proteins are incorporated, are also involved in many biological processes such as signal transmission and ATP synthesis. Both proteins and lipids are dynamic objects, which are constantly fluctuating under the influence of the thermal energy (k_B_T = ~26 meV at T = 300 K; k_B_ is Boltzmann’s constant) of the surrounding environment, i.e., water in cells. It is now widely accepted that these molecules utilize thermal fluctuations to express their biological functions [3]. Whereas the dynamic nature of the molecules is essential for expressing proper functions, subtle changes in molecular properties such as the surrounding chemical environments or point mutations in the protein molecules or lipid composition of the cell membranes can cause a variety of diseases and disorders. It is therefore of critical importance to determine how the structural fluctuations of these molecules are related to not only their intrinsic biological functions, but also malfunctions leading to various diseases and disorders.

**Figure 1:**
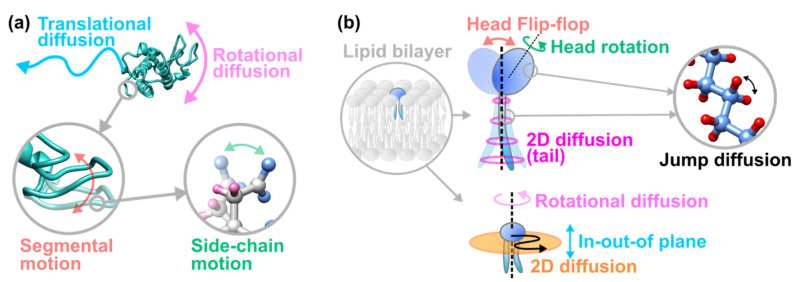
Hierarchical intramolecular motions observed by iNS for proteins (**a**) and lipid molecules (**b**). Classification of the motions of lipid molecules is based on the latest theoretical dynamical model called the Matryoshka model [15]. This figure is adapted from Ref. [16] with permission.

Incoherent neutron scattering (iNS) is one of the most powerful techniques to understand the dynamical behavior of bio-macromolecules. This technique resolves the structural fluctuations of target molecules at the ångström length scale and at the ps-ns timescale [4]. Furthermore, one of its advantages is that any form of samples such as solids (powders and crystals), solutions, or even cell pellets and biological tissues can be employed. Thermal fluctuations occurring at the ps-ns timescales cover a transition range in space and time from discrete localized motions at the fs timescale to collective large-scale motions taking place at the μs-ms timescale [5,6,7]. The representative motions of proteins and lipids observed by iNS are illustrated in Figure 1a,b, respectively. As shown in Figure 1, molecular dynamics show a hierarchy and as for proteins, various motions from localized side-chain motions, segmental motions corresponding to backbone motions, to global motions such as translational and rotational diffusions of a whole molecule can be observed. Regarding lipids, various classes of motions are present, from localized motions of the head group and the tail group of a lipid molecule, to global motions of the entire molecule. Thus, in both cases, hierarchical dynamics can be investigated from local atomic fluctuations to large-scale diffusive motions, which have widely been studied by iNS for more than three decades [8,9,10] since the pioneering and seminal works on the dynamics of the protein myoglobin by Doster et al. [11] and on the dynamics of DPPC lipid membranes by Pfeiffer et al. [12]. While the majority of studies have focused on the fundamental aspects of the dynamics of bio-macromolecules within the framework of molecular physics, some studies have sought to establish a possible relationship between the dynamical behavior of bio-macromolecules and their intrinsic biological functions. So far, excellent and comprehensive reviews on iNS studies featuring these aspects have been published [9,10,13,14]. On the other hand, there has emerged another research trend focusing on the relevance of bio-macromolecular dynamics to biological malfunctions, i.e., human diseases and disorders. Unlike conventional studies which have been performed mainly by physicists and chemists, this kind of research requires collaboration between physicists/chemists, biochemists, and physiologists. At the moment, there has been no literature review focusing on this emerging trend. We are therefore aiming to review the current circumstances in this field.

In this article, we focus on recent iNS studies on pathologically relevant proteins and lipids and discuss how the findings are of importance to elucidate the molecular mechanisms of human diseases and disorders that each study targets. The biological systems described in this review range from hydrated powder samples and solution samples of bio-macromolecules to cancer cells and brain tissues.

## 2. Basic Information on Incoherent Neutron Scattering

In this section, several pieces of information on iNS, which are required to follow this review, are introduced. For more details of the contents described in this section, readers are referred to other literature [4,9,17,18]. The scattering process between a neutron and a specimen is divided into two parts, coherent and incoherent scattering. The former is generally utilized for the structural analysis of molecules in crystallography or small-angle scattering. On the other hand, the iNS technique focuses on the latter. The incoherent neutron scattering cross-section of the hydrogen atom is much larger than that of any other atom constituting bio-macromolecules and its isotope deuterium [19]. Therefore, the iNS spectra are dominated by the contribution of hydrogen atoms in the samples. Since hydrogen atoms are quasi-uniformly distributed in space throughout bio-macromolecules, the dynamical information obtained by iNS provides the motions of hydrogen atoms averaged over the whole molecule.

The time scale (Δt) that is explored by iNS can be calculated by Heisenberg’s uncertainty principle,

(1) $$\Delta E\cdot \Delta t\geq \frac{\hbar }{2},$$

where ΔE (=ℏΔω, where ℏ (=1.055 × 10^−34^ J∙s) is the reduced Planck’s constant or Dirac’s constant and ω is the angular velocity) is the energy transfer of the scattered neutron with respect to the incident neutron. ΔE values intrinsic to neutron spectrometers are called energy resolution. Typically, ≅1–100 μeV of energy resolutions are employed in iNS measurements, which correspond to time resolutions (Δt) of ≅6.6–660 ps.

The iNS spectra S(Q, ω) are functions of momentum transfer Q (=4πsinθ/λ, where θ is half the scattering angle and λ is the wavelength of the neutron) and the energy transfer ω. Note that the energy transfer should be denoted by a symbol ΔE, but conventionally ω is often used as the energy transfer in the unit of ℏ.

Here we focus on two techniques included in iNS, which are elastic incoherent neutron scattering (EINS) and quasi-elastic neutron scattering (QENS). In EINS, the elastic scattering intensity S(Q, ±ΔE), i.e., the intensity integrated within the energy resolution, is analyzed. In the lower Q region, S(Q, ±ΔE) can be approximated by a Gaussian distribution [20]:

(2) $$S\left(Q,\;\pm \Delta E\right)=S\left(0,\pm \Delta E\right)\exp\left(-\frac{Q^{2}u^{2}}{3}\right),$$

where $\langle u^{2}\rangle$ is the mean square displacement (MSD) of H atoms. This approximation is valid under the condition $Q^{2}\langle u^{2}\rangle \leq 1$ [21] and can be extended to $Q^{2}\langle u^{2}\rangle <4.0$ for proteins [22]. Furthermore, from the temperature dependence of the MSD, the effective force constant or resilience 〈k〉 can be extracted:

(3) $$\langle k\rangle =\frac{k_{B}}{\frac{du^{2}}{dT}},$$

where *k_B_* is Boltzmann’s constant. The effective force constant is a measure of molecular flexibility, where larger 〈k〉 values mean more rigid molecules and vice versa.

**Figure 2:**
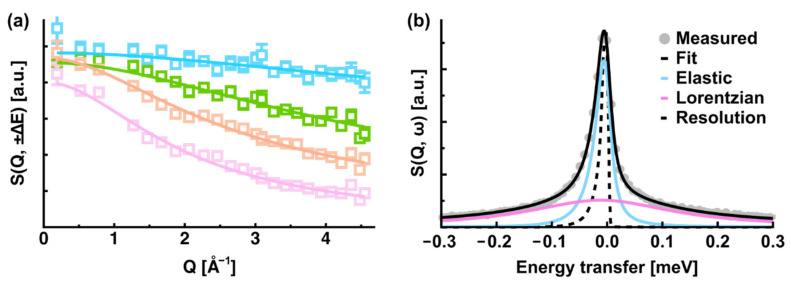
(**a**) Examples of EINS curves as a function of Q. Experimental data are shown in open squares while the solid lines denote the fits using Equation (4) in the main text. The data of lysozyme amyloid fibrils taken at 96 K, 200 K, 252 K, and 295 K are shown in cyan, green, orange, and pink, respectively [26]. (**b**) Example of the fitting of the QENS spectra. The resolution function is denoted by a broken line. The elastic and quasi-elastic components are shown in cyan and magenta, respectively. This fit corresponds to n = 1 in Equation (6).

The above Gaussian approximation is valid only in the low-Q region. The analysis using the entire Q-range can be performed using an analytical model taking into account the non-Gaussian behavior. The latest model in this line [23] describes the S(Q, ±ΔE) as

(4) $$S\left(Q,\;\pm \Delta E\right)=S\left(0,\pm \Delta E\right){\left(1+\frac{Q^{2}r^{2}}{\beta }\right)}^{-\beta },$$

(5) $$p\left(\lambda ,\beta \right)=\frac{\beta \exp\left(-\beta \lambda \right){\left(\beta \lambda \right)}^{\beta -1}}{\Gamma \left(\beta \right)}\left(0<\beta <\infty \right),$$

where $\langle r^{2}\rangle \;$ is the mean-square atomic position fluctuation (MSPF) of atoms. $p\left(\lambda ,\beta \right)$ describes the distribution of the atomic position fluctuations, which is based on a Gamma distribution. β describes a measure of motional heterogeneity. λ= $r^{2}/\langle r^{2}\rangle$ represents the squared atomic position fluctuation with respect to the MSPF. As easily seen, the Gaussian approximation (Equation (2)) is retrieved in the limit of β→ ∞. Examples of the fitting of the EINS curves using this model are shown in Figure 2a, where EINS curves of hydrated protein powder samples taken at different temperatures are displayed.

In QENS, other dynamical parameters mainly related to the rate of motions are obtained. The experimental QENS spectra S(Q, ω) contain scattering contributions from all the hydrogen atoms in the molecules and hence it is not possible to deconvolute them into individual contributions. Instead, a phenomenological fitting is employed to analyze the iNS spectra as follows:

(6) $$S\left(Q,\;\omega \right)=C\left(Q\right)\left[A_{0}\left(Q\right)\delta \left(\omega \right)+\sum_{i=1}^{n}A_{i}\left(Q\right)L_{i}\left(Q,\;\omega \right)\right]⨂L_{G}\left(Q,\;\omega \right)⨂R\left(Q,\;\omega \right)+B\left(Q\right)$$

where $C\left(Q\right)\;$ is the scaling factor between the experimental and the simulated spectra and includes the Debye–Waller factor denoting the atomic vibrations. The terms in [] represent local atomic motions such as side chains and $L_{G}\left(Q,\;\omega \right)$ denotes global motions occurring at a much slower time scale than the first term. ⨂ is the convolution operator. ${\;A}_{0}\left(Q\right)\delta \left(\omega \right)$ is the elastic scattering component. $A_{0}\left(Q\right)$ is called the elastic incoherent structure factor (EISF) and $A_{i}\left(Q\right)$ is called the quasi-elastic incoherent structure factor (QISF), which provides information on the geometry of motions. $R\left(Q,\;\omega \right)$ is the resolution function, which can be obtained by a vanadium measurement, and $B\left(Q\right)$ is the background brought about by the fast vibrations of atoms and/or the experimental setup. The terms $\sum_{i=1}^{n}A_{i}\left(Q\right)L_{i}\left(Q,\;\omega \right)$ are the quasi-elastic components and each component is described by a Lorentzian function ${\;L}_{i}\left(Q,\;\omega \right)$:

(7) $$L_{i}\left(Q,\;\omega \right)=\frac{1}{\pi }\frac{\Gamma_{i}\left(Q\right)}{\omega^{2}+\Gamma_{i}{\left(Q\right)}^{2}},$$

where $\Gamma_{i}\left(Q\right)$ is the half width at half maximum (HWHM) of the i-th Lorentzian function. The amplitudes $A_{i}\left(Q\right)$ are normalized under the condition $\;\sum_{i=0}^{n}A_{i}\left(Q\right)=1$. $L_{G}\left(Q,\;\omega \right)$ is also a Lorentzian function with the width of $\Gamma_{G}\left(Q\right)$. In the case of free diffusion, a global diffusion coefficient (D_G_) can be obtained by the relation $D_{G}=\Gamma_{G}\left(Q\right)/Q^{2}$ [4]. While the value of n depends on the systems, in many cases it is 1–3. An example of a fitting of the QENS spectra is shown in Figure 2b, where the QENS spectra of proteins in solution are fitted using Equation (6) with n = 1. While Equation (6) assumes that the local motions and the global motions are dynamically coupled, the term $L_{G}\left(Q,\;\omega \right)$ can be omitted depending on the samples investigated, e.g., in the case of hydrated powder samples, where global diffusion is suppressed.

From the Q^2^ dependence of the width of a Lorentzian function, several dynamical parameters are obtained with the help of the jump diffusion model [24]:

(8) $$\Gamma_{i}\left(Q\right)=\frac{D_{\mathrm{jump}}Q^{2}}{1+D_{\mathrm{jump}}Q^{2}\tau_{\mathrm{jump}}},$$

where D_jump_ is the jump diffusion coefficient and τ_jump_ is the residence time of the jump diffusion.

Regarding the analysis of the EISF, the diffusion-inside-a-sphere model (the Volino model) [25] is often employed:

(9) $$A_{0}\left(Q\right)=\left(1-{\;p}_{0}\right){\left(\frac{3j_{1}\left(\mathrm{Qa}\right)}{\mathrm{Qa}}\right)}^{2}+{\;p}_{0},$$

where ${\;p}_{0}$ is the fraction of “immobile” atoms, the motion of which is so slow that it is seen as fixed within the energy (time) resolution employed. $j_{1}\left(\mathrm{Qa}\right)$ is the first-order spherical Bessel function of the first kind. In this model, it is assumed that atoms can move within a sphere and a in Equation (9) is the radius of the sphere.

The above dynamical parameters are often referred to throughout this article to discuss the results of each study. Below, recent iNS studies on bio-macromolecules that are relevant to human diseases and disorders are reviewed.

## 3. iNS Studies on Pathologically Relevant Bio-Macromolecules

### 3.1. Protein Mutants as a Cause of Cardiomyopathy

**Figure 3:**
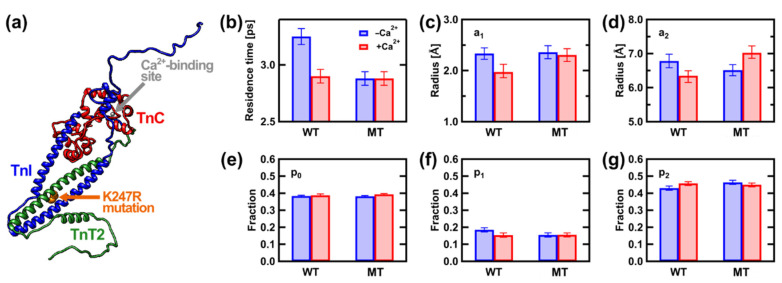
(**a**) Structure of Tn-CD. This model was generated based on a crystal structure (PDB ID: 4y99) with missing regions modeled as described elsewhere [37]. The protein structures in this paper are displayed using UCSF Chimera [43]. (**b**–**g**) Summary of the dynamical parameters obtained. The values in the absence and presence of Ca^2+^ are shown by blue and red bars, respectively. The EISF analysis was conducted assuming two mobile fractions atoms (p_1_ and p_2_) moving within spheres of radii a_1_ and a_2_, respectively, in addition to the immobile fraction p_0_ based on Equation (9) in the main text. Comparisons of the residence time (**b**), the values of the small amplitudes (**c**) and those of the large amplitudes (**d**), the immobile fraction of atoms (**e**), the fraction of the atoms moving with the small amplitude (**f**), and the fraction of the atoms moving with the large amplitude (**g**) are shown. These figures were adapted from Ref. [35] with permission.

Hereditary disease is one common form of human diseases. Familial cardiomyopathy is one of such diseases and is mainly classified into three types called hypertrophic cardiomyopathy (HCM), dilated cardiomyopathy (DCM), and restrictive cardio myopathy (RCM). There are many mutations in various proteins in cardiac muscles, which cause cardiomyopathy, and one of the major cardiac proteins is troponin (Tn), which consists of three sub-units TnC, TnI, and TnT [27]. Tn is a calcium-binding protein and forms fibrillar supramolecular complexes called “thin filaments”, together with an actin filament (F-actin) and tropomyosin. Ca^2+^ binding to TnC induces a series of structural changes in TnI and TnT, which displaces tropomyosin around F-actin, partially exposing the binding sites of F-actin to another protein myosin. Subsequent interactions between myosin and actin generate contractile forces in cardiac muscles. Tn thus plays a regulatory role in cardiac muscle contraction and is often called a regulatory protein. The functionally important domain of Tn is called the troponin core domain (Tn-CD), which consists of TnC, TnI, and TnT2 (part of TnT) (Figure 3a).

There are more than 60 mutations identified as causes of HCM, DCM, and RCM [28]. Among these, point mutations of K247R and E244D in TnT2 are known as causes of HCM and cause functional aberration such that the maximum force developed by the cardiac muscles increases without changing the Ca^2+^ binding sensitivity to TnC [29,30,31,32]. However, the molecular mechanism of the functional aberration remains unclear. The fact that cardiomyopathy-causing mutations are often located in the flexible regions of Tn [31], which constitute ~30% of the total residues and are not resolved by X-ray crystallography [33], implies that molecular flexibility plays an important role in the pathogenesis of familial cardiomyopathy.

The effects of the K247R mutation on the molecular dynamics of Tn-CD were investigated by QENS using the backscattering spectrometer BL02 DNA [34] at J-PARC/MLF (ΔE = 12 μeV) in Japan at 300 K [35]. As specimens, D_2_O solution samples of the wild-type Tn-CD (WT) and the K247R mutant (MT) both in the presence and absence of Ca^2+^ were used. Regarding the global motions, it was found that the observed global diffusion coefficients (D_G_) were larger than those calculated by HYDROPRO [36] based on the structural models derived from small-angle X-ray scattering (SAXS) [37]. The fact that the rigid body motions such as the translational and rotational diffusions are insufficient to reproduce the observed D_G_ values indicates that these values contain contributions from large-scale intramolecular motions such as segmental motions, as observed and demonstrated in several other studies [38,39,40]. It was found that the segmental motions of Tn-CD are not affected by Ca^2+^ binding nor by the mutation.

As for local motions, the residence time of the atomic motions decreased from 3.25 ± 0.07 ps to 2.88 ± 0.06 ps by the mutation in the absence of Ca^2+^ while the amplitudes of the atomic motions remained unchanged (the dynamical parameters obtained are summarized in Figure 3b–g). Since force generation is inhibited in both cardiac muscles containing the WT and the MT, the regulatory function of Tn-CD should not be affected by the mutation in the absence of Ca^2+^. The above result implies that the change in amplitudes may be required to modulate the function of Tn-CD. Upon Ca^2+^ binding, the residence time decreased to 2.91 ± 0.06 ps in the WT with a tendency to decrease the amplitudes. These dynamical changes are those that are essential to express the normal regulatory function. On the other hand, upon Ca^2+^ binding, the MT showed the unchanged residence time and significantly increased amplitude. The increased amplitude is in line with the increased MSD for the K247R mutant as obtained by an EINS measurement [41]. These results suggest that the molecular flexibility of the Tn-CD increases by the mutation and that the atoms in the MT can search a larger conformational space than those in the WT in the presence of Ca^2+^. It is likely that the MT with higher flexibility cannot keep tropomyosin in an appropriate position to control the actin–myosin interactions. A later study using small-angle X-ray scattering showed that tropomyosin is indeed displaced further by the E244D mutation, which has the same functional aberration as K247R, in the direction where force production is promoted, which is the functional aberration of this mutant [42]. These findings show that local atomic motions occurring at a sub-nanosecond timescale are relevant to the molecular events leading to the pathogenesis of HCM, which emphasizes the importance of the intramolecular motions accessible by iNS.

### 3.2. Proteins Related to Amyloidosis

**Figure 4:**
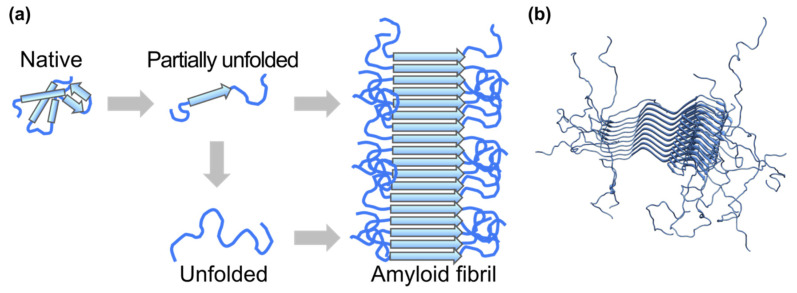
(**a**) Schematic illustration of amyloid fibril formation. (**b**) Atomic structure of amyloid fibrils of α-synuclein solved by solid state NMR (PDB ID: 2N0A). The ordered “core” region and the outside flexible regions are seen.

Diseases such as Alzheimer’s disease, Parkinson’s disease, and type-II diabetes are classified as amyloidosis, where the deposition of protein aggregates called amyloid fibrils is the common hallmark [44]. Protein monomers in (partially) unfolded states aggregate into amyloid fibrils, which are rich in β-sheets (Figure 4a). This suggests that the flexible nature of monomers plays an important role in the amyloid fibrillation processes. The arrangements of the β-strands in amyloid fibrils are called cross-β structures, where β-strands run in the direction perpendicular to the fiber axis [45]. Outside the “core” region of amyloid fibrils, consisting of the cross-β regions, are flexible regions which do not take on specific secondary structures [46] (Figure 4b). Interactions between fibrils and cellular or organelle membranes are considered one of the key processes causing cytotoxicity or neurotoxicity leading to the pathogenesis of amyloidosis. Since the outermost flexible regions of fibrils are the first regions that initiate the interaction and direct contact with lipid molecules, it is likely that the molecular flexibility of amyloid fibrils plays a crucial role in the pathogenesis of amyloidosis. Therefore, understanding the dynamical behavior of amyloid fibrils is essential for the elucidation of the molecular mechanism of amyloidosis.

**Figure 5:**
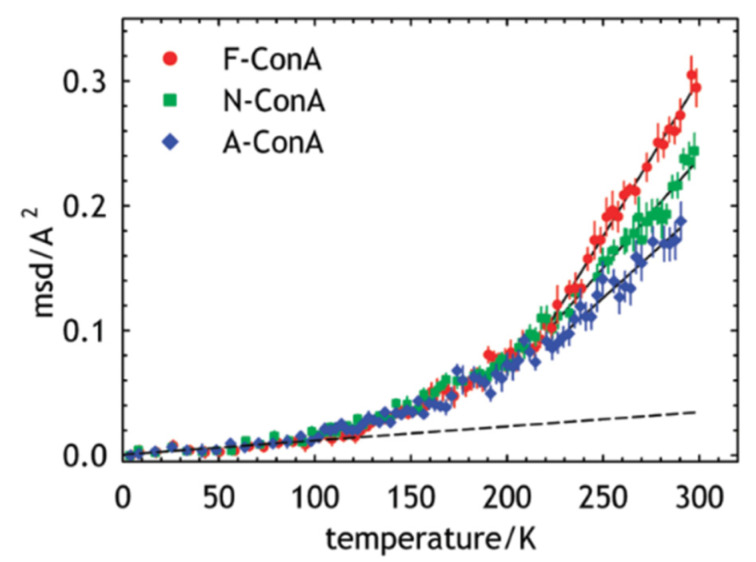
Comparison of the MSDs of concanavalin-A fibrils (F-ConA), monomers (N-ConA), and amorphous aggregates (A-ConA). Dotted lines show the fits to the data points at lower temperatures. Solid lines at higher temperatures are the linear fits to estimate the effective force constants. Reprinted with permission from Ref. [47]. Copyright 2022 American Chemical Society.

The first neutron scattering study on amyloid fibrils was reported in 2012 [47]. In this study, the dynamics of monomers, amorphous aggregates, and amyloid fibrils of concanavalin A (ConA), an amyloid model protein, was investigated using EINS and QENS at the backscattering spectrometer IN16 [48] of Institut Laue-Langevin (ILL) in France, with an energy resolution of 0.9 μeV. Powder samples hydrated with D_2_O (0.2 g D_2_O/g ConA) were employed as specimens to minimize the solvent contribution to the scattering signal. Since the labile hydrogen atoms of protein molecules are exchanged with the deuterium of the solvent, the observed dynamics by iNS reflects the motions of non-exchangeable hydrogen atoms. From the EINS measurements, the MSD of the fibrils was found to be larger than that of the other two samples, as shown in Figure 5, while the effective force constant was much smaller for the fibrils, suggesting that ConA fibrils show enhanced dynamics compared with monomers and amorphous aggregates. The EISF analysis of the QENS data also corroborated this result. This study thus challenged the paradigm that a more ordered structure is less flexible at least for local side chain motions, pointing to a complex relation between protein structure and dynamics. The higher flexibility of the ConA fibrils was attributed to side chains exposed to the fibril surface during amyloid fibrillation, which reduces the steric hindrance from other amino acid residues [47]. The increase in MSD upon fibrillation has also been observed for lysozyme amyloid fibrils in water/ethanol mixtures [49].

**Figure 6:**
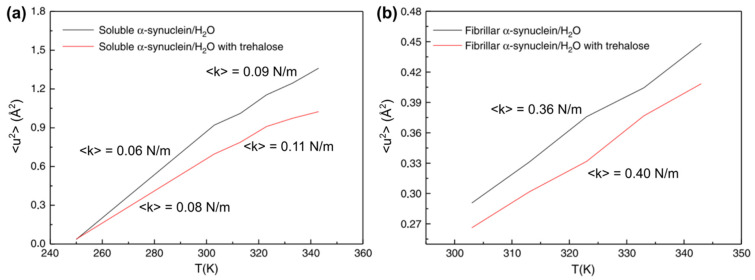
Comparison of the MSDs (<u^2^>) and the effective force constants (<k>) between (**a**) monomeric (soluble) α-synuclein with and without trehalose, and between (**b**) fibrillar α-synuclein with and without trehalose. Note that the data shown in (**a**,**b**) were measured using different spectrometers and thus direct comparison between (**a**,**b**) is not possible. This figure was reproduced from Ref. [50] with permission.

**Figure 7:**
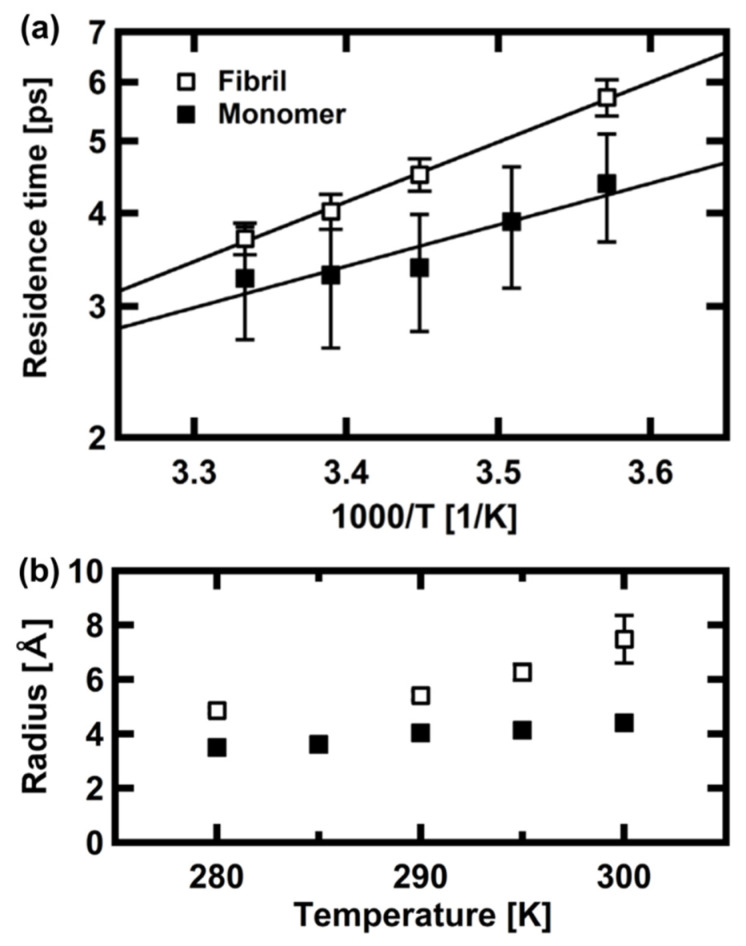
Dynamical parameters of α-synuclein monomers and fibrils in solution. (**a**) Residence times as a function of the inverse of temperature. (**b**) Radii of the sphere estimated by the EISF analysis using a diffusion-inside-a-sphere model. Filled and open squares denote the monomers and fibrils, respectively. This figure was reproduced from Ref. [39].

Later, the molecular dynamics of the monomers and fibrils of α-synuclein, which is the causative agent of Parkinson’s disease, have been characterized by QENS [50]. In order to see the effects of bio-protectant and thermal stress on the α-synuclein dynamics, QENS measurements were carried out with/without trehalose either at 250–343 K using the OSIRIS spectrometer (ΔE = 99 μeV) [51] or at 303–343 K using the IRIS spectrometer (ΔE = 16 μeV) [52] of the Rutherford Appleton Laboratory in the UK. The analysis of the elastic component of the QENS spectra measured at OSIRIS showed that, as shown in Figure 6a, the effective force constant increases from 0.06 N/m (below 303 K) to 0.09 N/m (above 303 K) in the absence of trehalose, pointing to a conformational change of α-synuclein monomers. Trehalose increased the above force constants by 0.02 N/m, suggesting its stabilizing effect on protein fluctuations. The QENS data taken at IRIS revealed that, as shown in Figure 6b, the effective force constant of the α-synuclein fibrils increases from 0.36 N/m to 0.4 N/m by the addition of trehalose, indicating again the stabilizing effect of trehalose. Note that a direct comparison of the force constants between monomers and fibrils cannot be made because of the different energy resolutions. The direct comparison of the sub-nanosecond dynamics of α-synuclein monomers and fibrils by QENS was performed using BL02 DNA [34] at J-PARC/MLF (ΔE = 12 μeV) on D_2_O solution samples [39]. It was found that whereas the residence times of local motions are larger for fibrils (3.5–5.5 ps between 280 K and 300 K) than for monomers (3–4 ps) (Figure 7a), the radius of a sphere obtained by the EISF analysis was larger for fibrils (5–7 Å between 280 K and 300 K) than for monomers (~4 Å) (Figure 7b), suggesting that the amplitudes of the side chain motions are larger in fibrils than in monomers. The higher flexibility of the α-synuclein fibrils is consistent with the findings of the above studies. These results have been interpreted to mean that there exists solvent space within the fibrils, facilitating the side chain motions of the fibrils. In relation to this interpretation, enhanced hydration water mobility around the tau amyloid fibrils compared with tau monomers has been reported [53], implying the importance of water molecules in amyloid formation.

Amyloid fibrillation strongly depends on the solvent conditions such as the pH, temperature, and salt concentrations. Regarding α-synuclein, it is known that the incubation of α-synuclein at a neutral pH leads to the formation of fibrils while incubation at an acidic pH leads to amorphous aggregates consisting of short fibrils [54]. In order to study the dynamical properties of α-synuclein in relation to different propensities of fibril formation, QENS measurements were carried out on D_2_O solution samples of α-synuclein monomers at pD 4.0, pD 7.4 with 150 mM of NaCl, and pD 7.4 without salt using BL02 DNA as above [55]. At pD 7.4, fibril formation is significantly accelerated with 150 mM of NaCl compared with the no salt condition. From the analysis of global motions, it was found that the segmental motions were the largest for pD 7.4 with NaCl while the other two samples showed a similar degree of segmental motions. As for local motions, the residence times of the samples of pD 7.4 with NaCl and of pD 4.0 were similar (3–7 ps between 280 K and 299 K) and were smaller than that of pD 7.4 without salt. The radius of the sphere obtained by the EISF analysis showed that the radius was the largest (6–7 Å) for pD 4.0 above 290 K while that for pD 7.4 with salt and for pD 7.4 without salt were the second largest (~5 Å) and the smallest (~4.5 Å), respectively. Furthermore, a structural analysis of α-synuclein monomers by small-angle X-ray scattering (SAXS) in the same study showed that monomers at pH 7.4 with NaCl were expanded while the other two samples took compact conformations. Together with other lines of structural evidence, these models were interpreted to mean that at pH 7.4 with NaCl, the N- and C-terminal regions of α-synuclein are expanded, while at the other two conditions, the N- and C-terminal regions are located closely to the NAC region (the central region). The combined results of SAXS and QENS suggest that the enhancement of both the local and the segmental motions is required for fibril formation while the enhancement of only the local motions induces non-specific interactions, leading to amorphous aggregates. This study indicates that the morphological properties of aggregates are controlled by the balance between small-scale local atomic motions and large-scale segmental motions.

**Figure 8:**
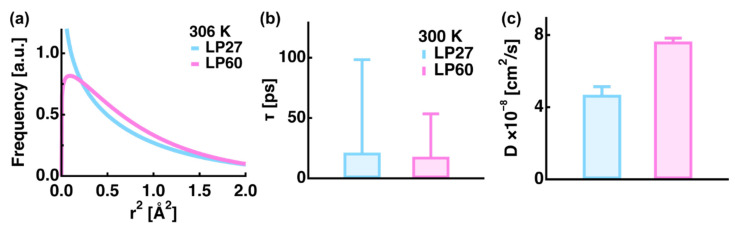
Comparison of the dynamical parameters of lysozyme amyloid polymorphs called LP27 and LP60. (**a**) Distribution of amplitudes of local atomic motions calculated using Equation (5) in the main text. (**b**,**c**) Residence times (**b**) and the jump diffusion coefficients (**c**) of lysozyme polymorphs obtained by QENS. In all the panels, the data of LP27 and LP60 are denoted in cyan and magenta, respectively. This figure was reproduced from Ref. [26].

All the above studies focus on the process of amyloid fibril formation. In addition to this, an intriguing property of the amyloid system is its polymorphism, where the same protein forms fibrils with different morphologies and physicochemical properties depending on fibrillation conditions [56]. Since different characteristics of polymorphs are considered to be correlated with the degree of cytotoxicity, disease duration, and disease severity, it is of critical importance to reveal the structural and dynamical natures of amyloid polymorphs. It has been shown that human and hen egg white lysozyme amyloid fibrils formed at a near neutral pH are more cytotoxic than those formed at an acidic pH [57,58]. The former is an aggregate of short fibrils and the latter is an unbranched long fibril [58]. Moreover, a recent study has demonstrated that the former has the ability to bind monomers much more than the latter, which leads the authors to hypothesize that the lysozyme amyloid polymorphs formed at a near neutral pH may undergo fast configurational diffusion with lower energy barriers [59]. In order to investigate the possible dynamical differences between these polymorphs, EINS and QENS measurements were carried out at the backscattering spectrometer IN13 [60] at ILL (ΔE = 8 μeV) on hydrated powder samples (0.4 g D_2_O/g protein) of two kinds of amyloid polymorphs of hen egg white lysozyme formed at pH 2.7 (LP27) and at pH 6.0 (LP60) [26]. An analysis of the EINS data revealed that while the MSPF values were the same for both samples, the distribution of the atomic position fluctuations was largely different: LP60 contained a larger fraction of atoms undergoing diffusive motions with larger amplitudes than LP27 (Figure 8a). Moreover, an analysis of the QENS spectra showed that the jump diffusion coefficient of the atoms of LP60 (7.6 ± 0.2 × 10^−8^ cm^2^/s) was significantly larger than that of LP27 (4.7 ± 0.4 × 10^−8^ cm^2^/s) while the residence times were similar within errors, as shown in Figure 8b,c. These results suggest that LP60 has a higher molecular flexibility than LP27, which supports the recent hypothesis denoted above [59]. These findings indicate that LP60 has the ability to take much more conformations than LP27, which facilitates binding and interactions with other molecules. This property would promote monomer binding in polymorphs formed at a near neutral pH [59]. At the same time, since interactions between amyloid fibrils and cellular or organelle membranes are recognized to be important in causing cytotoxicity [61], the more dynamic LP60 might be able to bind to bio-membranes more easily than LP27, leading to stronger membrane disruption and hence to stronger cytotoxicity for LP60 [58]. Future EINS and QENS studies will reveal the details of the dynamical nature of amyloid polymorphs interacting with bio-membranes to give deeper insights into the molecular mechanism of cytotoxicity, and eventually amyloidosis.

### 3.3. Model System Mimicking Myelin

In our nervous systems, i.e., the central nervous system (CNS) and the peripheral nervous system (PNS), nerve transmission is accelerated by myelin, which takes a multi-lamellar membrane structure by winding around nerve axons. PNS myelin consists of four types of proteins: the myelin basic protein (MBP), the P0 glycoprotein, the myelin protein 2 (P2), and the peripheral myelin protein-22, in addition to lipids such as phospholipids and cholesterol. Several point mutations in P2 have been identified as causes of Charcot–Marie–Tooth (CMT) disease, which is one of the hereditary human neuropathies and modulates nerve transmission in both motor and sensory nerves in the PNS [62]. Furthermore, a lack of MBP causes primarily demyelinating disease [63]. Since lipid membranes are characterized by their dynamic fluidity, an understanding of the molecular dynamics of lipids and their changes caused by the insertion of relevant proteins, as well as the dynamics of myelin-constituting proteins, is important to reveal the molecular mechanism of the myelin function and its relevant diseases.

**Figure 9:**
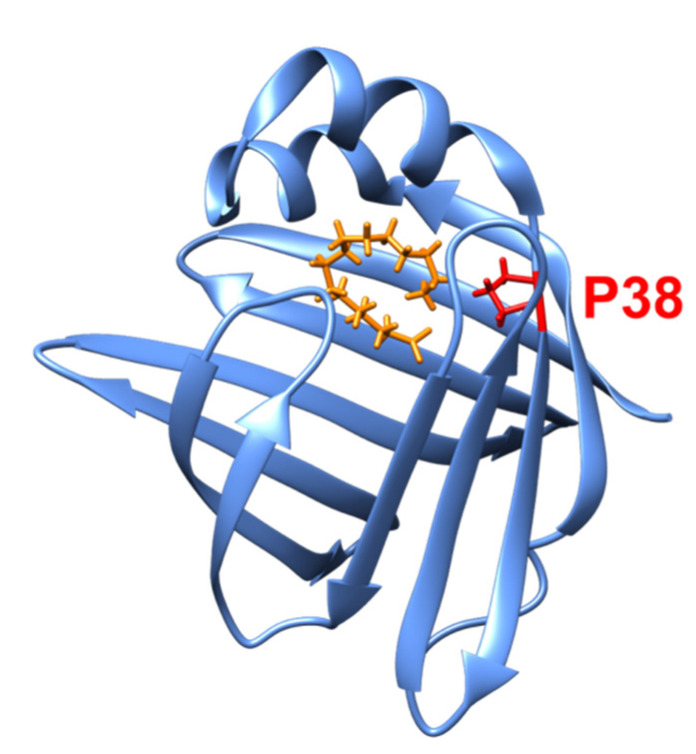
Crystal structure of a myelin protein P2 (PDB ID: 7O60). Two helices form the “lid” of the molecule. Shown in orange is a palmitic acid and P38 is shown in red.

**Figure 10:**
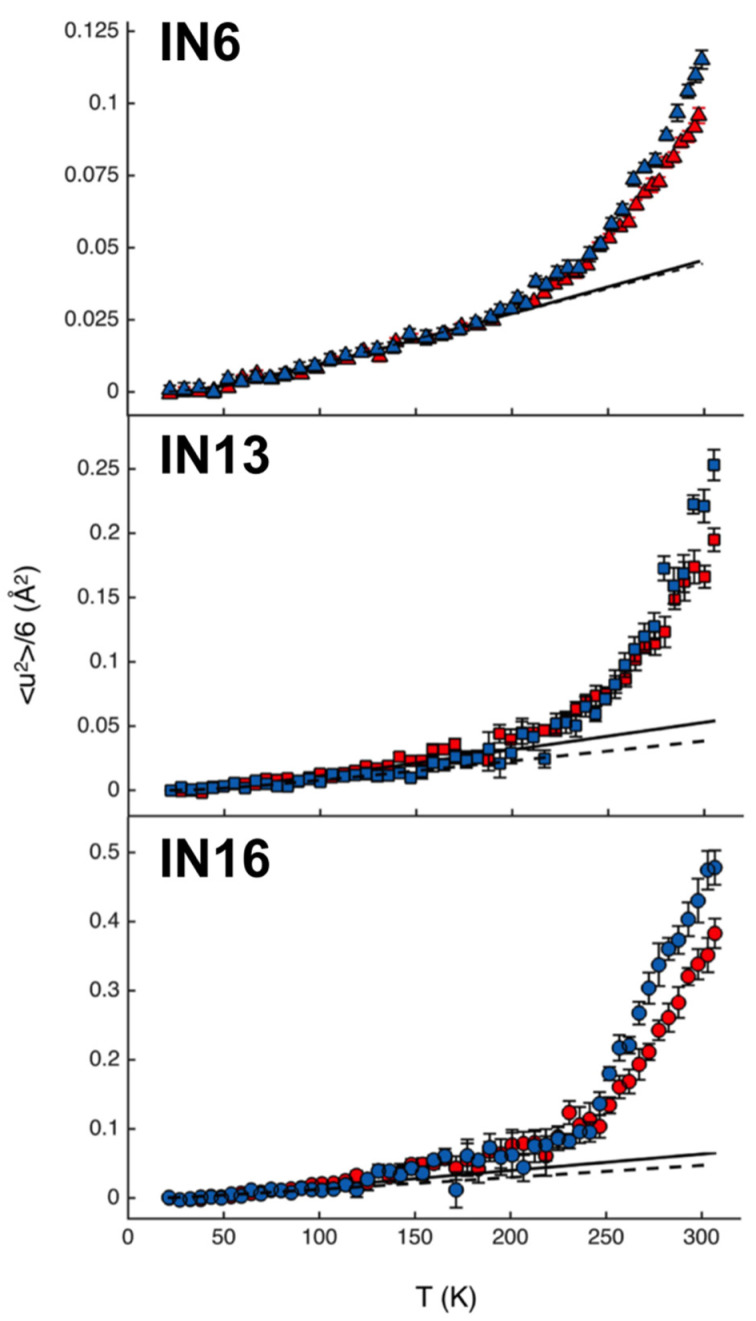
Comparison of the MSDs of P2 protein as a function of temperature. Note that the longitude is represented as <u^2^> divided by six. The data of the wild-type P2 and the P38G mutant are denoted by red and blue, respectively. The solid and the dotted lines are fits in the low temperature regions. This figure was reproduced from Ref. [65].

Regarding myelin proteins, the effects of a point mutation (P38G) on the molecular dynamics of P2 have been studied in relation to their functional changes using EINS on hydrated powder samples [64,65]. P2 is a 15 kDa protein and has a fatty acid binding pocket between the lid and the β-barrel structure (Figure 9), which plays a role in fatty acid transport and homeostasis in myelin [66]. Although this mutation, which is located in the hinge region between the lid and the β-barrel, is not one of the causes of CMT disease, the P38G mutant increases the lipid-binding activity [64]. EINS measurements on IN6 (ΔE = 70 μeV) [67], IN13 (ΔE = 8 μeV) [60], and IN16 (ΔE = 0.9 μeV) [48] at ILL showed that the MSD of the P38G mutant is larger than that of the wild-type P2 by 0.05–0.2 Å^2^ at 300 K as shown in Figure 10 [65]. In addition, the effective force constant estimated above 250 K decreased from 0.46 N/m to 0.32 N/m by the mutation [64], suggesting that the mutant has a higher molecular flexibility than its wild-type counterpart and that increased molecular flexibility enhances the lipid-binding activity.

As for the membrane dynamics, Knoll et al. [68] used EINS to investigate the effects of the insertion of P2 on the dynamics of lipids in unilamellar vesicles, which consist of 1,2-dimyristoyl-*sn*-glycero-3-phosphate (DMPA) or a binary mixture of 1,2-dioleoyl-*sn*-glycero-3-phosphocholine (DOPC) and 1,2-dioleoyl-*sn*-glycero-3-phospho-L-serine (DOPS). In the temperature range employed (278 K < T < 313 K), the DMPA liposomes and the DOPC-DOPS liposomes are in the gel and the liquid phases, respectively. Low P2 concentration of 1.25% of the total mass are allowed to focus on membrane dynamics. EINS measurements were carried out on IN13 [60] at ILL with an energy resolution of 8 μeV. It was demonstrated that the addition of P2 increases the effective force constant from 0.036 N/m to 0.053 N/m (for DMPA liposomes), and from 0.018 N/m to 0.023 N/m (for DOPC-DOPS liposomes), suggesting that the human P2 protein stabilizes the lipid membrane.

**Figure 11:**
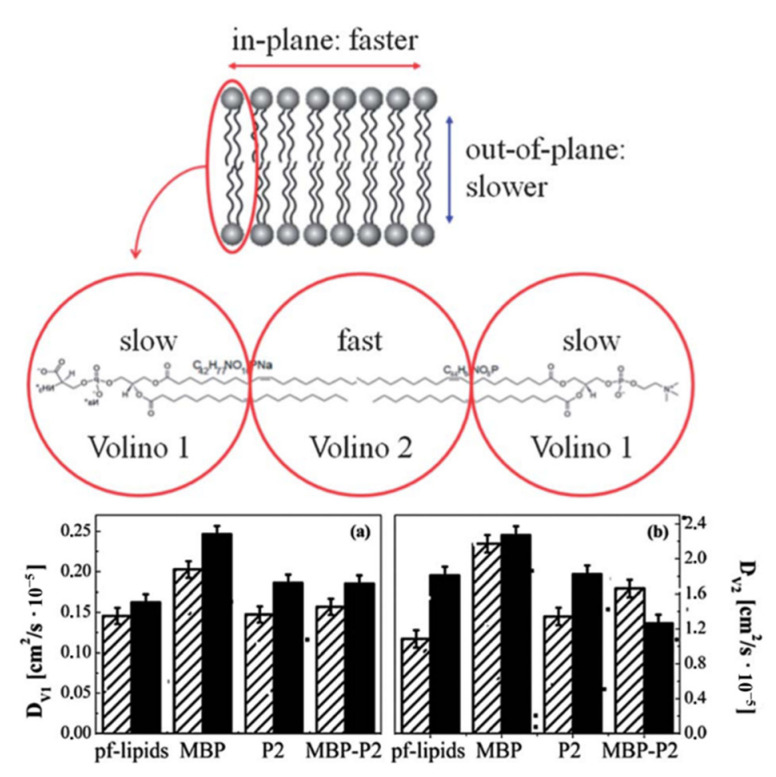
(**Top**): schematic of lipid bilayers and assignment of the Volino model to different parts of the lipid molecules. (**Bottom**): diffusion coefficients D_V1_ of the region “Volino 1” (**a**) and D_V2_ of the region “Volino 2” (**b**). Half-filled bars and filled bars represent out-of-plane and in-plane configurations, respectively. pf-lipids denotes protein-free lipids. This figure was reproduced from Ref. [69].

Later, the structural and dynamical changes of DOPC-DOPS bilayers caused by the addition of P2, MBP, or both P2 and MBP were studied using neutron diffraction and QENS [69]. Neutron diffraction data taken on D16 [70] at ILL showed that the addition of proteins changed the structure of the protein-inserted membranes: the inter-bilayer distances changed from 59 Å (the protein-free bilayers) to 59–79 Å for the mixture depending on the protein species. This result was interpreted as the effect of major regions being structurally affected by proteins whereas other minor regions remained in the protein-free state. Considering the fact that the fraction of the membrane surface of the protein-bound lipids is in the range of 0.2%–0.5% (the protein concentration is half of that of the study described above [68]), this result shows that protein binding has a long-range effect on the membrane structure. As for the dynamics of lipids, an analysis of the QENS spectra measured on IN5 [71] at ILL (ΔE = 12 μeV), on Osiris [51] at ISIS (ΔE = 100 μeV), and on Neat [72] at Helmholtz-Zentrum Berlin (ΔE = 216 μeV) based on a theoretical dynamical model, which assigns a diffusion-inside-a-sphere model (the Volino model) to both the head and the tail groups of lipid molecules (Figure 11), showed that the diffusion coefficients of the region both closer to the head group (D_v1_) and closer to the tail group (D_v2_) increased by the addition of MBP, suggesting that the lipid molecules undergo enhanced diffusive motions in MBP-inserted membranes. On the other hand, the insertion of P2 was shown not to affect both D_v1_ and D_v2_. The loss of the membrane structure as seen by neutron diffraction could enhance the lipid dynamics, but the authors explain that this potential enhancement of dynamics is compensated for by reduced dynamics caused by a strong interaction between positively charged P2 and negatively charged lipids. In the case of the addition of both P2 and MBP, the diffusion coefficient D_v1_ was found to remain unaffected. On the other hand, while D_v2_ in the in-plane direction was found to decrease, that in the out-of-plane direction increased, reflecting the fact that complex interactions between proteins and lipids, which include the deep penetration of MBP into bilayers, result in the opposite change of the diffusion coefficients of the region closer to the tail of the lipid molecules.

The above studies on the model myelin membranes have thus revealed quantitatively how the dynamics of the model membrane mimicking myelin are affected by myelin-constituting proteins depending on protein species and the number ratio of protein molecules to lipids. Since MBP and P2 are considered to be responsible for the compaction and stability of myelin structure, the dynamical information discussed above will be useful to understand the molecular mechanism of the structural integrity of myelin sheath and diseases such as CMT disease.

### 3.4. Low-Density Lipoprotein in Terms of Pathology

Low-density lipoproteins (LDL) transport lipids such as cholesterol and triglyceride to cells and thus are essential macromolecular assemblies for homeostasis. LDL consists of various lipids and a single protein called apolipoprotein B-100 (apo B-100). The surface of LDL is covered by a phospholipid monolayer and its hydrophobic core contains cholesteryl esters and triglycerides. Apo B-100 plays an important role in the LDL functions through the stabilization of the structure of the lipid assemblies and involvement in the binding to cellular lipoprotein receptors. It is known that the accumulation of oxidized LDLs in the vessel sub-endothelial space is the first stage of atherosclerosis. This disease is a risk factor of ischemic stroke, which is one of the major causes of mortality in the world. Furthermore, LDL particles with triglycerides rich in its hydrophobic core are found in patients suffering from hyperlipidemia. Therefore, the investigation of the dynamical behavior of oxidized LDL and triglyceride-rich LDL particles is crucial to gain insights into the mechanism of LDL-related diseases.

**Figure 12:**
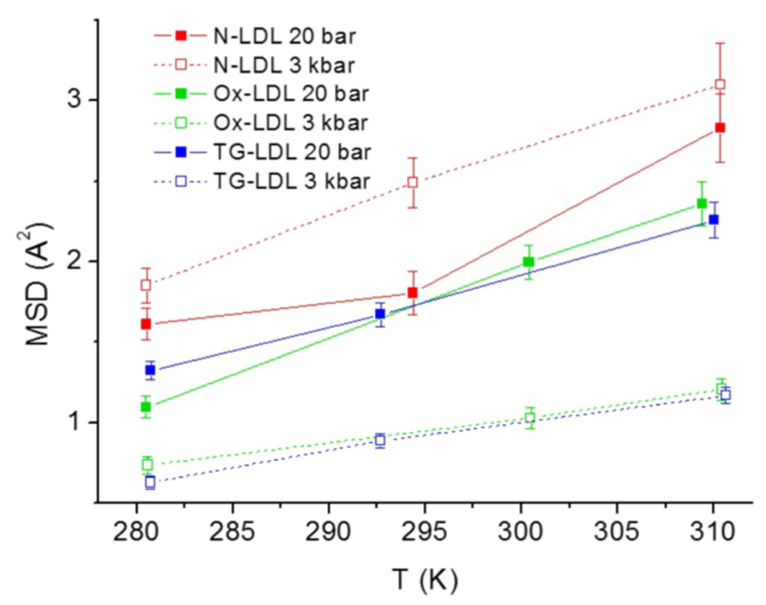
Comparison of MSD values extracted from EINS data of normal LDL (N-LDL), TG-LDL, and Ox-LDL as a function of temperature at 20 bar and 3000 bar [73]. This figure was reproduced with kind permission from The European Physical Journal (EPJ E).

A comparison of the sub-nanosecond dynamics of normal LDL, triglyceride-rich LDL (TG-LDL), and oxidized LDL (Ox-LDL) was made using EINS coupled with high hydrostatic pressure (HHP) equipment [73]. The EINS measurements were carried out on IN13 (ΔE = 8 μeV) [60] at ILL on D_2_O solution samples of the above three types of LDL between 280 K and 310 K at two pressure points of 20 bar and 3 kbar. As shown in Figure 12, the resultant MSD values were found to increase with temperature, as was expected for all the three LDLs. On the other hand, the dynamical response to HHP showed unique features: at the higher pressure, the MSD values decreased for both LDLs mimicking pathological conditions while those of the normal LDL remained similar to those at a lower pressure within errors, except for 295 K, which is close to the phase transition temperature of 297 K. These results suggest that the normal LDL has the ability to cope with HHP in terms of molecular dynamics whereas LDLs in pathological forms do not. The above results were interpreted as follows [73]: Triglycerides are much softer than cholesteryl esters and their quantity is enhanced in the lipid core of TG-LDL. Furthermore, a higher triglyceride content leads to a lower phase transition temperature. These factors are considered to cause reduced atomic motions at higher pressures. Regarding Ox-LDL, the oxidation of LDL is known to destabilize the protein moiety (apo B-100) with little effects on the core lipid organization [74]. In addition, the oxidation of the acyl chains of the phospholipids in the monolayer comprising the surface of LDL leads to the less densely packing of lipid molecules. These factors are considered to be a cause of higher compressibility at a higher pressure. Revealing how the modified dynamics of LDL impact LDL accumulation in blood vessels will be key to understand the molecular basis of atherosclerosis.

The above study also suggests the importance of using HHP for the investigation of the dynamical behavior of bio-macromolecules: temperature has a similar effect on all the samples (increase in MSDs with increasing temperature) whereas pressure induces dynamical responses unique to each sample depending on its molecular compressibility. Therefore, temperature and pressure are the two most important thermodynamic variables to fully understand the physical properties of the target system.

In relation to this study on LDL, the characterization of apo B-100 has recently been conducted using QENS [75]. Since apo B-100 is an amphiphilic protein, the use of detergents called Nonidet P-40 (NP40) was necessary to solubilize the protein. In this study, hydrated powder samples of apo B-100/NP40 complexes at 0.4 g D_2_O/g sample were used as specimen and their QENS spectra were measured using IN5 (ΔE = 10 μeV) [71], IN16B (ΔE = 4 μeV), and IN16B-BATS (ΔE = 0.75 μeV) [76] at ILL in order to analyze the dynamics in wide time windows. The use of multiple energy resolutions permitted to separate the contributions of apo B-100 and NP40 to the QENS spectra. An analysis of the QENS spectra showed that the residence time of the domain motions of apo B-100 (550 ps) was significantly larger than that of NP40 (67 ps) at ΔE = 0.75 μeV. The jump diffusion coefficients of local atomic motions were also different between apo B-100 (1.3 Å^2^/ns) and NP40 (9.5 Å^2^/ns). Furthermore, an analysis of EISF and QISFs showed that the radius of confinement was smaller for apo B-100 (3.2 Å) than for NP40 (4.6 Å) at the same energy resolution. These results suggest that in apo B-100/NP40 complexes, the domain motions of apo B-100 are restricted by interactions with NP40. Since the internal dynamics and functions of membrane proteins depend on the type of surrounding lipids or detergents [77], the above method of dynamical analysis will be useful to observe, for example, the dynamics of apo B-100 in normal LDL and in a pathological form of LDL containing oxidated lipids.

In the dynamical analysis of apo B-100 in complex with NP40, a recently published dynamical model called the Matryoshka model [15,16,78] was employed with some modifications. The Matryoshka model takes into account various kinds of intramolecular motions manifested in phospholipids in addition to the diffusion of the entire molecule. This model will thus demonstrate its power to obtain detailed dynamical information on systems such as pathologically relevant lipids or lipid–protein complexes in combination with protein perdeuteration.

### 3.5. Brain Tissue and Cancer Cell

In addition to the bio-molecular solutions or powder samples described above, the application of iNS has now been expanded to biological tissues such as the brain in terms of pathology. From diffusion magnetic resonance imaging (*d*MRI), it has been shown that water diffusion is modulated in some diseases such as ischemia and tumors [79]. Since this technique provides dynamical information at micrometer and tens of millisecond scales, the detailed water mobility cannot be accessed through this technique. In this respect, iNS serves as a complementary technique and contributes to draw a physical picture on the molecular events taking place in brains in healthy and pathological states.

Natali et al. used QENS to investigate water dynamics in tissue sections of the cerebral right hemisphere (RH) of a bovine brain [80]. QENS measurements were carried out on tissue sections fixated by formalin (RH_fixed_) and those dealt with cryo-protectants (liquid nitrogen) (RH_cryo_) using IN5 (ΔE = 10 μeV) [71] at ILL at two temperatures, 250 K and 300 K. These two techniques are often used for the preservation of biological tissues and organs. Note that it was confirmed that the long exposure (2 h) of RH_cryo_ at 300 K does not cause any change in terms of proton dynamics. The QENS spectra at 300 K were analyzed assuming two populations of water molecules, which are free (faster motions) or restricted (slower motions). The existence of the two populations was later confirmed by simultaneous measurements by *d*MRI and QENS [81]. It was found that the two preservation protocols had different effects on the mobility of water molecules in both populations: Whereas the translational diffusion coefficient of the free water molecules was the same within the errors for both protocols (2.5 ± 0.7 × 10^−5^ cm^2^/s and 2.5 ± 0.3 × 10^−5^ cm^2^/s for RH_fixed_ and RH_cryo_, respectively), the residence time of RH_fixed_ (2.6 ± 0.3 ps) was larger than that of RH_cryo_ (1.8 ± 0.4 ps). In addition, the translational diffusion coefficient of the restricted water molecules of RH_fixed_ (0.17 ± 0.02 × 10^−5^ cm^2^/s) was smaller than that of RH_cryo_ (0.25 ± 0.03 × 10^−5^ cm^2^/s) while the residence time was larger for RH_fixed_ (6.4 ± 0.1 ps) than for RH_cryo_ (5.7 ± 0.3 ps). These results suggest that water dynamics are reduced by formalin fixation, which is known to form a disordered network structure through the cross-linking of proteins. Water molecules may be trapped in this disordered network, leading to restricted dynamics. On the other hand, at a much lower temperature of 250 K, opposite effects were observed [80]. The QENS spectra at this temperature were fitted assuming an elastic and one Lorentzian component due to the reduction in the water dynamics at the freezing temperature. An analysis of EISF showed that the radius of confinement of RH_fixed_ (5.2 ± 0.3 Å) was much larger than that of RH_cryo_ (1.9 ± 0.5 Å), suggesting that the water molecules could move in a larger space in RH_fixed_. This enhancement in water dynamics was interpreted as being caused by supercooling. The fixation methods thus significantly affected the water dynamics in brain tissues. Therefore, when investigating molecular behavior in ex vivo brain tissues, which often requires fixation, some cautions have to be taken. The quantification of the dynamical parameters of water molecules as described above will help to characterize such samples correctly.

**Figure 13:**
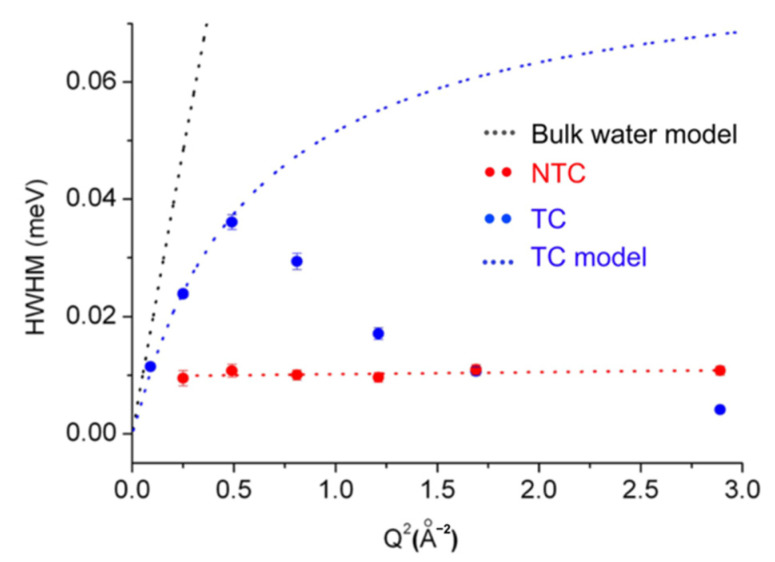
Comparison of the HWHM values of a Lorentzian function obtained by the fitting of the QENS spectra of PTX-treated cells (TC) and non-treated cells (NTC). The black dotted line denotes a theoretical curve for bulk water obtained from the jump diffusion model where the diffusion coefficient and the residence time were 2.9 × 10^−5^ cm^2^/s and 1 ps, respectively. The fit to the TC data by the jump diffusion model is described by the blue dotted curve. The red dotted line represents a linear fit of the NTC data points. This figure was reproduced from Ref. [82].

Another example of a study on biological tissues is the one on water dynamics in cancer cells using QENS [82]. Water dynamics were investigated on human breast cancer cells (MCF-7) which were treated (hereafter called TC) or non-treated (NTC) with anti-cancer drug paclitaxel (PTX). QENS measurements were carried out using BASIS (ΔE = 3.5 μeV) at SNS [83] in the USA. The spectra were analyzed by a single Lorentzian, the width of which is shown for both samples in Figure 13. It was shown that while the HWHM of NTC was Q-independent, indicating the localized motions such as rotational motions, that of TC showed a Q-dependent behavior, which was analyzed by the jump-diffusion model. Note that the HWHM values of TC showed a drastic decrease as Q increased (Figure 13). This is interpreted by motions that are so fast that the broadening of the Lorentzian cannot be fitted correctly. At much larger energy resolutions such as 30 μeV, the motions of bulk water as well as hydration water can be captured (see for example, [84]). An analysis of the HWHMs in Figure 8 showed that the treatment of cancer cells with PTX enhances intracellular water dynamics so that the translational diffusion coefficient and the residence time are 2.2 ± 0.1 × 10^−5^ cm^2^/s and 9.1 ± 0.6 ps, respectively. While the former is close to bulk water, the latter is much larger than bulk water, suggesting that the intracellular water molecules interact with other molecules. The findings obtained by this study will be useful to promote our understanding of drug behavior inside living cells. In order to further characterize the intracellular water dynamics in a pathological context, it would be necessary to employ various cell lines and distinct chemotherapy agents, as suggested by the authors [82], as well as to arrest the cells in each of the different phases of the cell cycle because the internal architecture of the cells depends on the phase. In this regard, a very recent QENS study investigated water dynamics in different types of cancer cells, which were breast and tongue cancers [85]. This study has clearly demonstrated that water mobility strongly depends on cancer types: breast cancer tissues contain more mobile water fractions than the corresponding normal tissues while the opposite is observed for tongue cancer tissues. On the other hand, there were no significant dynamical differences between non-tumor specimens. This study strongly suggests that water dynamics are sensitive to cancer types and hence would serve as a bio-marker for cancer diagnosis as well as a key component to understand the molecular mechanism of cancer pathogenesis.

## 4. Future Perspective

In pathologically relevant systems, iNS has been utilized to identify changes in dynamical parameters describing the amplitudes and frequency of atomic motions and thus the chemical groups to which they are bound, in response to pathological perturbations such as aggregation, mutations, and chemical modifications. These dynamical changes are interpreted by making use of findings obtained from other experimental techniques and/or simulations. In this way, molecular pictures containing not only information on structure but also on molecular flexibility have been drawn for several target systems in relation to relevant functional modulation or dysfunction, leading to the advancement of our understanding of the molecular mechanism of various diseases and disorders.

On the other hand, the inherent nature of iNS, which provides dynamical information averaged over all the hydrogen atoms in the bio-macromolecule, makes it almost impossible to locate the regions which are affected by pathological perturbations. The assignment of the dynamical parameters extracted from iNS studies onto protein or lipid structures will be key to obtain further information on atomic details. One possible way to overcome the loss of spatial information is to use NMR, MD simulation, or normal mode analysis. This combination has already been employed in several systems (for example, [86]), but it is not a prevalent way to interpret the iNS data at the moment because of the size and complexity of the target bio-macromolecules. The constant improvement of the performance of both supercomputers and personal computers will make this approach more common. Another possibility to increase the spatial information is to use partial deuteration, where part of a bio-macromolecule is deuterated while other parts are in a hydrogenated state. This enables one to extract dynamical information from only the hydrogenated part of the molecules. This technique has already been used for iNS studies on phospholipid molecules, but it is not common in iNS studies on proteins because of the technical difficulty of producing such samples.

An advantageous point of iNS is that it allows any form of samples to be studied, which allows much larger biological systems to be studied. For example, the investigation of the macromolecular dynamics in droplets formed by liquid–liquid phase separation (LLPS) will be useful to shed light on the molecular mechanism of diseases such as the amyotrophic lateral sclerosis (ALS) or of the coronavirus replication cycle [87,88]. Compared with bio-macromolecules, cells are extremely large and intracellular organizations are complicated. For such complex systems, even the use of current supercomputers to obtain agreements between iNS experiments and simulations might not be feasible within a reasonable time frame unless using coarse-grained approaches [Di Bari et al., submitted]. Nevertheless, the quantification of water mobility in cells with/without potential or commercially available drugs is still invaluable to obtain clues about molecular interactions and the architecture of the local environment where the water molecules reside. Furthermore, the dynamical characterization will make it possible to design drug molecules that show desirable diffusive motions inside cells. The ability to characterize atomic motions at the ångström length scale and at the ps-ns timescale makes iNS a unique probe to investigate the dynamical behavior of bio-macromolecules not only in a physiological state but also in a pathological state. Combined with other complementary techniques covering length and time scales that iNS does not cover, this technique will help elucidate the molecular mechanisms of various human diseases by seamlessly linking relevant information from the atomic level to cellular and tissue levels. Since some diseases such as amyloidosis have become more relevant in the aging society, research in this field will continue to develop further and to be more important amid the current increasing trend for longevity worldwide.

## Data Availability

Not applicable.
